# The Hydrophobic Stabilization of *Pseudomonas aeruginosa* Bacteriophage F8 and the Influence of Modified Bacteriophage Preparation on Biofilm Degradation

**DOI:** 10.1007/s00284-024-03896-2

**Published:** 2024-09-22

**Authors:** Bożena Szermer-Olearnik, Karolina Filik-Matyjaszczyk, Jarosław Ciekot, Anna Czarny

**Affiliations:** Hirszfeld Institute of Immunology and Experimental Therapy, Polish Academy of Sciences, 12 R. Weigl St, 53114 Wroclaw, Poland

## Abstract

The bacteriophage F8 belongs to the *Myoviridae* group of phages and is a pathogen of *Pseudomonas aeruginosa*. Since *Pseudomonas aeruginosa* is a multidrug-resistant opportunistic bacterium and can cause serious challenges for health services, studying the potential use of phages against them is a promising approach. *Pseudomonas aeruginosa* can be found on medical devices because bacteria can attach to surfaces and develop biofilms, which are difficult to eradicate because of their high resistance to environmental conditions and antimicrobial therapeutics. Phage therapy is becoming promising as an alternative for the treatment of antibiotic-resistant infections, but there is still a lack of standardized protocols approved by health organizations for possible use in the clinic. In our research, we focused on the potential use of 1-octanol, which was previously used by our team to develop a method for phage purification from bacterial lysate. 1-octanol is a fatty alcohol that is mostly used in the cosmetics industry, and its advantage is that it is approved by the FDA as a food additive. In this paper, we studied the protective properties of the addition of 1-octanol for storing phage liquid preparations. We demonstrated the stabilization effect of 1-octanol addition on F8 bacteriophage preparation during storage under various conditions. Interestingly, more effective biofilm reduction was observed after treatment with the purified bacteriophage and with 1-octanol addition compared to crude lysate.

**Supplementary Information:**

The online version contains supplementary material available at 10.1007/s00284-024-03896-2.

## Introduction

Bacterial resistance to antibiotics has become a major problem in modern medicine. ESKAPE organisms, which include *Pseudomonas aeruginosa*, have developed widespread resistance to antimicrobial agents [1] and are a serious challenge for health services. *Pseudomonas aeruginosa* is a multidrug-resistant (MDR) opportunistic pathogen that causes serious health problems in people with cystic fibrosis, cancer, or burns. In addition, the pathogen causes hospital-acquired infections. *Pseudomonas aeruginosa* can be found on medical devices because it can adhere to wet surfaces and develop a biofilm. Treatments for infections caused by these bacteria are challenging due to their rapid mutation and resistance to antibiotics [2, 3]. Many virulence factors of *P. aeruginosa* enable it to survive in the host organism. The most important are (I) lipopolysaccharide (LPS), which has toxic properties and is responsible for tissue damage, attachment to the surface, and host recognition; (II) outer membrane proteins (OMPs), which mostly participate in nutrient transport and adhesion; (III) flagella and pili, which participate in biofilm formation; (IV) six types of secretion systems, which function is the colonization of the host, adhesion, and chemotactic signaling; and (V) exopolysaccharides, such as alginate, Psl, and Pel, which may help facilitate biofilm formation [3, 4]. According to the Annual Epidemiological Report for the year 2020–2029, EU/EEA countries reported 20 675 isolates of *P. aeruginosa*. A total of 30.1% of the *P. aeruginosa* isolates reported to EARS-Net for 2020 were resistant to at least one of the antimicrobial groups under surveillance (piperacillin-tazobactam, fluoroquinolones, ceftazidime, aminoglycosides, and carbapenems). At the global level, the WHO has listed carbapenem-resistant *P. aeruginosa* as a critical priority pathogen, and research and the development of new antibiotics are needed [5, 6].

The widespread use of antibiotics and the slow development of effective antimicrobial agents have motivated scientists to develop novel drugs to treat infections caused by MDR strains [3]. The antibiotic resistance crisis and the search for alternative clinical treatments for bacterial infections can create favorable conditions for regulatory guidance standards for introducing phage therapy into clinical trials. Phages are ubiquitous and abundant in the biosphere. They are also a part of the human microbiome, which means that they are well tolerated in the human body and could be used successfully in therapy [7]. These viruses specifically infect bacteria and cause lysis of their cells. Moreover, phages are natural and specific antimicrobial agents because they can increase their numbers by infecting (with self-dose at the infection site) and producing virion progeny without disturbing the overall microbiome.

Personalized phage therapy is an interesting approach for treating patients with infections caused by MDR strains [8]. Prior to their use in therapy, it is essential to characterize bacteriophages biologically (burst size and life cycle), assess their preparation composition, evaluate their stability in body fluids, and add stabilizers to the final phage products. Many different factors should be included in the development of phage-based therapeutics, especially physicochemical aspects such as pH, temperature, and ions, as well as host physiological conditions and bacterial susceptibility to phages [8, 9]. The immune system is not neutral for bacteriophages and can reduce their concentration when there are no specific hosts in the environment [10]. The effects of adaptive immunity and antiphage antibody production on phages in the mammalian system are known. The response of the immune system seems to be dose-dependent, and high doses of phages can induce specific responses for long periods of time [7]. Hodyra-Stefaniak et al. indicated in a murine systemic inflammatory response syndrome (SIR) model that IgM and IgG responses inhibited F8 phages [11]. However, the immunogenicity of phages itself does not seem to be a significant safety risk for patients [7]. In addition to the immunological point of view, numerical simulations of phage–bacterium population dynamics show that this relationship seems to be an additional important factor for the success of therapy [12]. To conclude, it is important to deliver the right concentration of active bacteriophage to the infection site, and a key role is to develop a formulation that will maintain the phage titer at a high level for a certain period to remove all the pathogenic bacteria.

The production of phages is more complex than the production of chemical-based drugs. The main cause of this is the sensitivity of phages to production, purification, and storage conditions [13]. Purification is a high-risk process and could lead to the inactivation of the phage population in the first step. The significant point of purification is removing contaminants derived from the bacterial host, especially lipopolysaccharides (LPS). Defined phage formulation and stability are also critical factors for successful therapy. A series of papers describing the storage of bacteriophages in lyophilized form and deep freezing at -80 °C have been published [14–16]. Bacteriophage therapy is based mainly on the use of liquid formulations [17]. Duyvejonck et al*.* proposed an original method for bacteriophage stabilization in solutions commonly used in industry and concluded that a solution with stable and neutral pH and low ionic content was the best for liquid storage of the tested phages [18].

Previously, we designed a phage extraction method using 1-octanol. This method is efficient, inexpensive, and does not require usage of expensive equipment; moreover, it helps remove up to 10^5^ EU/mL (endotoxin unit) and maintain phage titer during the purification procedure [19]. At the same time, we observed that the use of 1-octanol allows not only efficient removal of endotoxins from aqueous bacteriophage lysates but also simultaneously preserving the infectivity of the bacteriophages. It was observed that trace residues of 1-octanol in the bacteriophage preparation after extraction allowed longer storage of the purified preparations while maintaining antibacterial activity. For this reason, we evaluated whether the addition of 1-octanol to the purified F8 phage solution could serve as a preservative for the long-term storage of this phage. 1-octanol is a fatty alcohol that occurs naturally in citrus oils and is successfully used in the perfume industry. Like ethanol, 1-octanol is metabolized by alcohol dehydrogenase and has been approved for human use as a food additive by the Food and Drug Administration (FDA) and the Council of Europe [20, 21]. 1-octanol was also studied in a pilot trial to treat essential tremor, a neurological disorder, and the results showed that the oral intake of a single dose of 1 mg/kg 1-octanol was well tolerated without signs of intoxication [20].

Important and required in the Quality Guidelines ICH from the point of stability and formulation studies is testing future drug substances in stress conditions such as defined temperature and humidity (Quality Guidelines ICH (Q1A-Q1F), WHO) [22]. Therefore, we decided to test received F8 bacteriophage preparations for their antibacterial activity in different temperature ranges. Additionally, more effective biofilm reduction was observed with the addition of purified bacteriophage and with 1-octanol addition compared to crude lysate. This is an interesting observation because of the high resistance of bacterial biofilms to eradication with antibacterial agents.

## Materials and Methods

### Bacteriophages and Bacterial Strains

*Pseudomonas aeruginosa* phage F8 and the host bacterium *Pseudomonas aeruginosa* PCM 2720 were obtained from the Polish Collection of Microorganisms, Institute of Immunology and Experimental Therapy, Polish Academy of Science, Wroclaw, Poland.

### Bacteriophage Propagation and Purification

An overnight bacterial culture (0.1 mL) was added to tubes containing 5 mL of Luria–Bertani (LB) broth and incubated at 37 °C with shaking to the exponential growth phase (OD 600 = 0.4–0.5). Then, 0.1 mL of crude phage lysate containing 2.6 × 10^8^ PFU/mL was added, and the mixture was incubated for 4 h at 37 °C with shaking and then left overnight at room temperature. The mixture was filtered through Millipore membranes with 0.22 µm-diameter pores to remove residual bacterial cells. The F8 bacteriophage was purified according to the procedure of Szermer-Olearnik and Boratyński [19].

### Determination of Endotoxin Levels

Endotoxin determination was performed using the LAL test (*Limulus Amebocyte Lysate*, Charles River Laboratories International). The quantification of endotoxins was achieved using the chromogenic technique. For each determination, a standard curve was generated. The samples were serially diluted with apyrogenic water until the measurement was within the range of the standard curve. Each data point was analyzed in triplicate. The reaction conditions were maintained according to the manufacturer’s recommendations.

### Incubation of Bacteriophage Preparations Under Different Conditions

The following preparations of the F8 bacteriophage were prepared: (1) lysate obtained after the multiplication of the F8 bacteriophage on *Pseudomonas aeruginosa* PCM 2720, (2) purified F8 bacteriophage obtained after using the extraction method described by Szermer-Olearnik and Boratyński [19], and (3) purified bacteriophage F8 with 1-octanol added at a final concentration of 100 mg/mL. The samples prepared in this way were stored at various temperatures, namely, − 20 °C, 4 °C, and 40 °C, and for different time points. Each sample was replicated three times. The titer of bacteriophages in the tested preparations was determined by the double-layer agar (DLA) technique.

### Electron Microscopy

The crude lysate and purified bacteriophage were applied to the surface of formvar carbon-coated copper grids and negatively stained with 2% uranyl acetate for 1 min. The excess uranyl acetate was then removed from the grids using filter paper, and the grids were allowed to air dry for 20 min. Preparations were visualized using a JEOL JEM-1200 EX 80 kV transmission electron microscope.

### Testing the Sensitivity of *Pseudomonas* aeruginosa PCM 2720 to Antibiotics

*Pseudomonas aeruginosa* PCM 2720 bacteria were grown in Luria–Bertani (LB) broth to the exponential growth phase (OD 600 = 0.4–0.5). After this, the 1 mL of bacteria suspension was poured onto each agar plate (Enrichment LAB-AGAR with enzymatic digest of casein, peptone, yeast extract, beef extract, sodium chloride) and spread evenly over the entire agar surface with a sterile spreader and finally dried at 37 °C for 40 min. Then, antibiotic disks were placed on the obtained bacterial layer and incubated overnight at 37 °C. Three antibiotics were used in the experiment: kanamycin (30 µg), colistin sulfate (50 µg), and ceftazidime (10 µg) (Oxoid Ltd., Basingstoke Hampshire England). The zone of inhibition of bacterial growth was assessed. The antibiotic that generated the largest diameter growth inhibition zone was selected for further study.

### Measuring the Effect on Biofilm Degradation

100 µL of inoculum containing 2.7 × 10^8^ CFU/mL *Pseudomonas aeruginosa* PCM 2720 was added to each well of 96-well plates and incubated for 24 h at 37 °C. The supernatant was collected, and 100 µL of control or bacteriophage sample was added to the formed biofilm. Control samples: LB, PBS, 100 mg/mL 1-octanol. Tested samples: F8 bacteriophage lysate (5.5 × 10^8^ PFU/mL), purified preparation of bacteriophage F8 (5.5 × 10^8^ PFU/mL), and purified preparation of bacteriophage F8 with 1-octanol (5.5 × 10^8^ PFU/mL). After 6 h of incubation of the preparations with the mature biofilm, the supernatant was removed, and the remaining biofilm was washed once with PBS, resuspended in 100 µL of PBS, and plated on agar plates to estimate the number of bacteria. Each sample was replicated three times. The statistical analysis was performed using one-way ANOVA with Tukey’s multiple comparison test.

In parallel, the same procedure for mature biofilms was performed using a light microscope slide Lab-Tek® Chamber SlideTM System (Thermo Fisher Scientific Inc. Rochester, NY, USA). The slides were rinsed with water, dried, and stained with 1% crystal violet. After 30 min, the slides were rinsed repeatedly with water and then observed under a light Olympus microscope (100×magnification).

### Stability of the Bacteriophage Preparation in Human Serum

The bacterial inoculum of an overnight bacterial culture was added to tubes containing 5 mL of Luria–Bertani (LB) broth and incubated at 37 °C with shaking to 0.5 McFarland. In the next step, 10 µL of bacterial suspension was added to a 96-well plate, 40 µL of LB medium, 50 µL of PBS, or 50 µL of bacteriophage preparation (the total volume per well was 100 µL at this stage of the experiment). Three types of phage preparation were used: F8 bacteriophage lysate (7.5 × 10^8^ PFU/mL), purified preparation of bacteriophage F8 (1.5 × 10^7^ PFU/mL), and purified preparation of bacteriophage F8 with 1-octanol (1.5 × 10^7^ PFU/mL). The plate was incubated for 2 h at 37 °C. After incubation in the indicated wells, 100 µL of active human-pooled serum (MP Biomedicals), 100 µL of inactive human-pooled serum (MP Biomedicals), or 100 µL of LB medium was added (ratio 1:1). Inactive serum was obtained by heating at 56 °C for 30 min. The plate was then incubated for an additional 2 h at 37 °C. After the indicated time points, the samples from the wells were diluted in PBS and plated on agar plates to calculate the CFU/mL. The agar plates were incubated for 24 h at 37 °C, after which the colonies were counted. Each sample on a 96-well plate was replicated three times. As a control, the following combinations were used: untreated bacterial cells, bacterial cells treated with phage preparations without active/inactive serum, bacterial cells treated with active/inactive serum but without phage addition, and bacterial cells treated with a 1-octanol solution with or without active/inactive serum addition. The tested samples were added to wells containing mixtures of phages and active serum. The statistical analysis was performed using one-way ANOVA with Tukey’s multiple comparison test.

## Results

### Preparation and Characterization of the Purified Bacteriophage F8

**Fig. 1:**
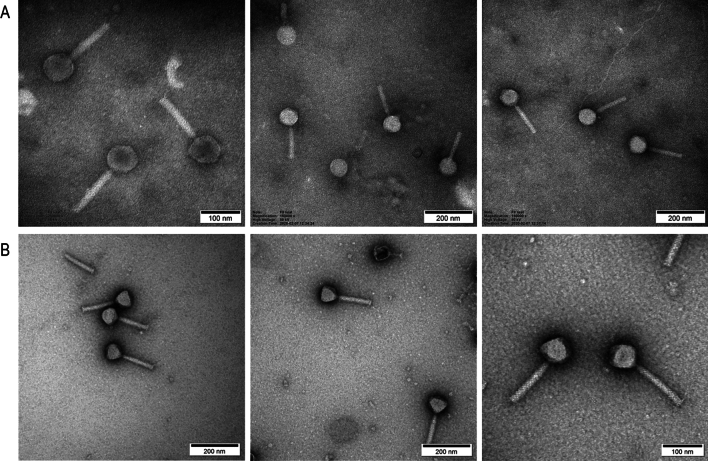
Transmission electron microscopy visualization of **A** crude bacteriophage F8 lysate and **B** bacteriophage preparation purified with a heterophasic extraction procedure

Crude bacteriophage F8 lysate was purified using a heterophasic extraction procedure according to Szermer-Olearnik et al. [19]. The purity of the obtained preparation was assessed using the LAL test to determine the endotoxin concentration, which was below 10 EU/mL. In addition, dynamic light scattering (DLS) measurements of the obtained purified preparation demonstrated its monodispersity (PdI value of 0.209) and average particle size of approximately 116.7 d.nm. DLS measurements and endotoxin level determination confirmed the high purity of the obtained F8 preparation (Fig. 1 sup). Additionally, the preservation of antibacterial activity in the preparation after purification was confirmed using the DLA technique. The crude lysate (Fig. 1A) and purified phage (Fig. 1B) were observed using transmission electron microscopy (TEM). The phage particles in the purified preparation had a visible capsid with sharp contours. The background of the photo (Fig. 1B) shows the elimination of many impurities relative to the crude lysate (Fig. 1A). Electron microscopy confirmed that the bacteriophage F8 belongs to the *Myoviridae* group and has a large capsid head and contractile tail. Based on the TEM images, it can be observed that the purified preparation allows for more detailed imaging of phage particles, which is an additional confirmation of the high purity of the obtained preparations.

### Sensitivity Testing of the Obtained Preparation Under Defined Conditions

**Fig. 2:**
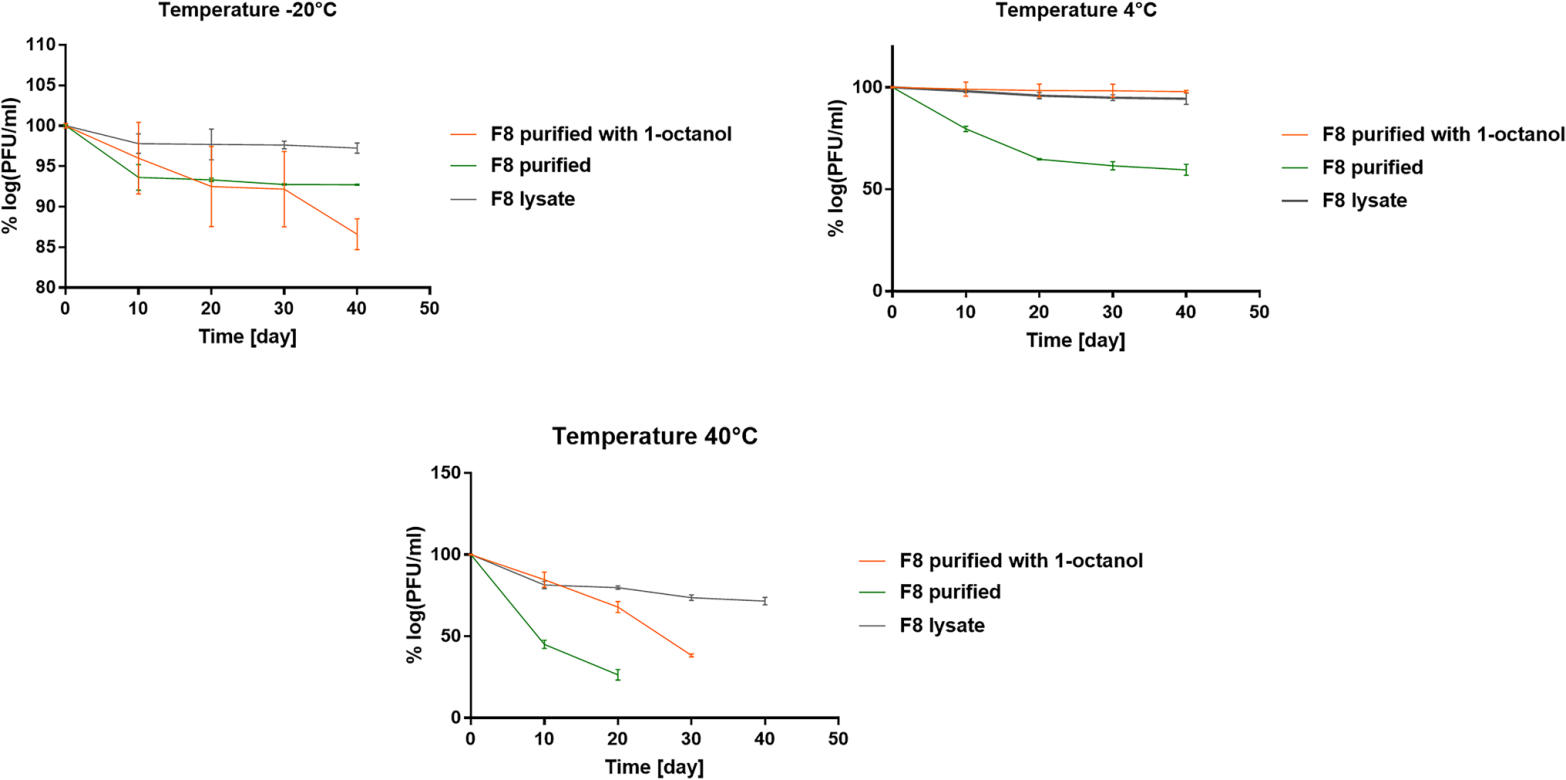
The graphs show the titer changes (expressed as percentages) of the F8 bacteriophage during storage at − 20 °C, 4 °C, and 40 °C (75% humidity). Crude bacteriophage F8 lysate—F8 lysate, purified preparation of bacteriophage F8—F8 purified, and purified preparation of bacteriophage F8 supplemented with 1-octanol—F8 purified with 1-octanol. The phage titers were determined by the DLA method

Next, the sensitivity of the obtained purified F8 bacteriophage preparation to storage conditions was tested, and the effect of the addition of 1-octanol on phage stability was assessed. For this purpose, the lytic ability of F8 bacteriophages against *Pseudomonas aeruginosa* PCM 2720 during storage under different temperature conditions was compared. As expected, the crude lysate was the most stable during long-term storage [23]. The purified preparation rapidly lost activity, but the addition of 1-octanol significantly improved the stability of the phage in the tested samples (Fig. 2). The results of testing the stability of phage preparation with the addition of 1-octanol in a climatic chamber under accelerated conditions (40 °C; 75% humidity) were interesting (Quality Guidelines ICH (Q1A-Q1F), WHO) because bacteriophage activity remained at the crude lysate level for almost 20 days in contrast to F8 purified without the addition of 1-octanol (Fig. 2).

### Testing the Sensitivity of *Pseudomonas* aeruginosa to Selected Antibiotics

The sensitivity of *Pseudomonas aeruginosa* PCM 2720 to selected antibiotics (kanamycin, colistin sulfate, and ceftazidime) was studied. The bacteria were sensitive to all of the selected antibiotics, and the inhibition zones measured for the individual antibiotics were as follows: kanamycin—8 mm, colistin sulfate—17 mm, and ceftazidime—20 mm. Ceftazidime, compared to the rest of the tested antibiotics, showed the largest inhibition zone of the tested *Pseudomonas aeruginosa* PCM 2720. This indicates the highest sensitivity of the tested strain to this antibiotic compared to the other two antibiotics. Therefore, ceftazidime was selected for further biofilm comparative studies as a control.

### Influence of the Obtained Preparations on Biofilm Degradation

**Fig. 3:**
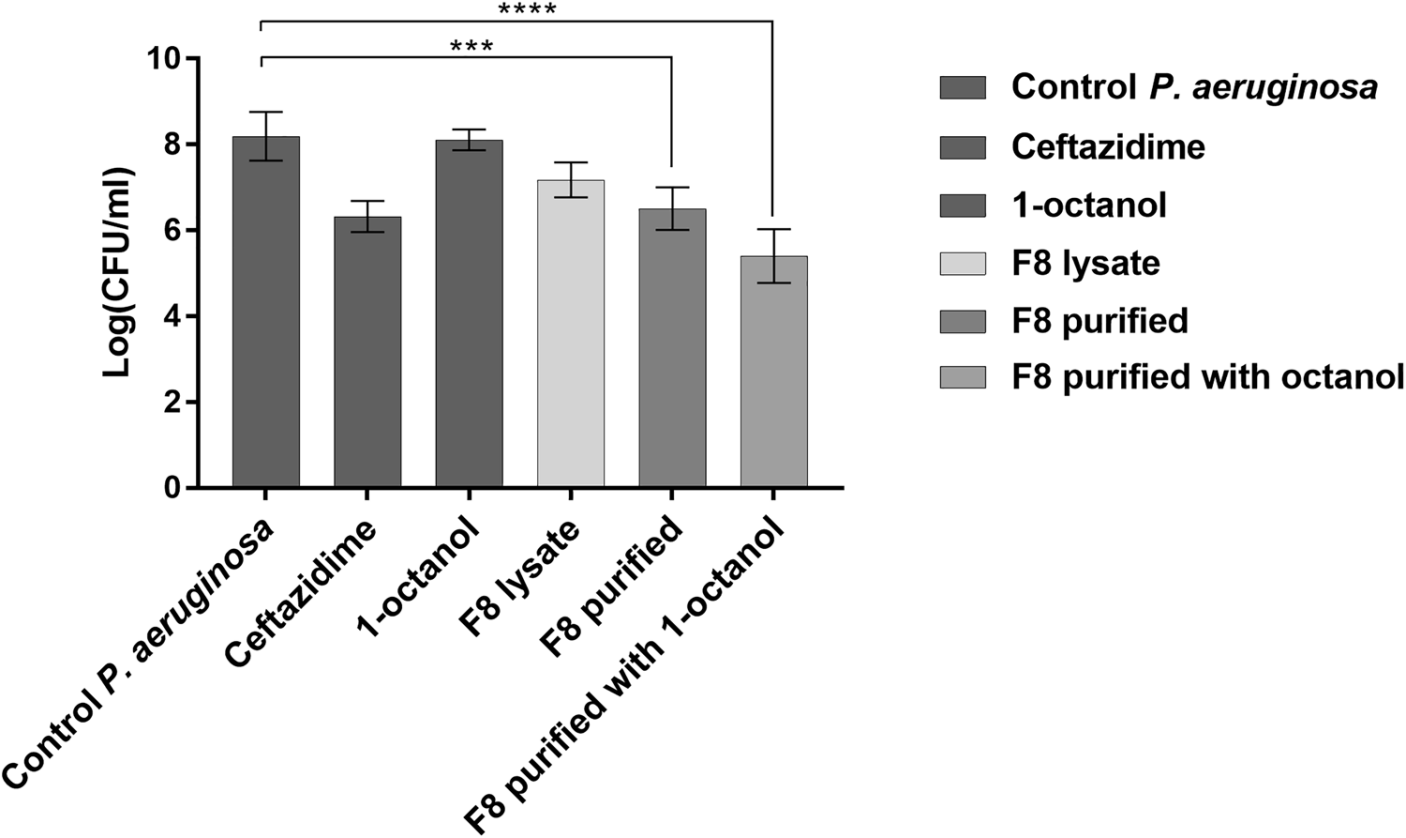
The graph represents the antibacterial activity of 3 forms of bacteriophage preparations (F8 lysate, purified F8 bacteriophage, and purified bacteriophage with the addition of 1-octanol) compared to that of the third-generation cephalosporin ceftazidime on biofilm degradation. A 1-octanol solution added alone to the bacteria was used as a control. ***p value < 0.005; ****p value < 0.0005. Antibacterial activity was determined by the culture plate method of the remaining biofilm

Next, the effectiveness of the different phage preparations on the control of biofilm was tested. Figure 3 shows the change in the number of bacteria after treatment with different F8 bacteriophage preparations compared to the untreated control. Among the tested phage preparations, the preparation purified with the addition of 1-octanol had the highest biofilm degradation activity. In addition, 1-octanol alone did not affect the number of bacteria. Differences in the number of bacteria after treatment of the biofilm with the purified preparation and purified preparation with the 1-octanol addition were statistically significant compared to the untreated control.

**Fig. 4:**
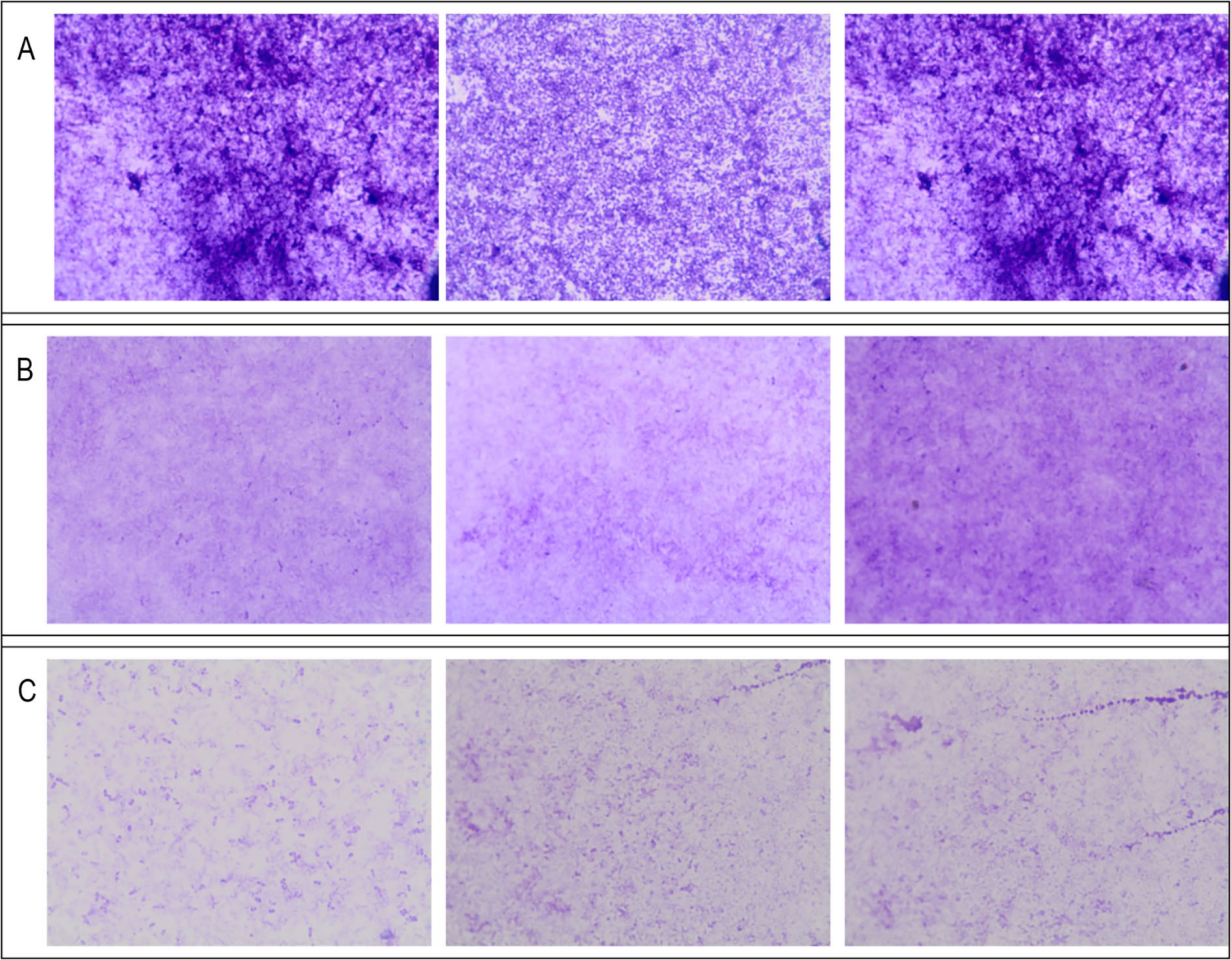
The effect of F8 bacteriophage preparations on the *Pseudomonas aeruginosa* PCM 2720 mature biofilm observed after crystal violet staining under an Olympus BX43 light microscope (magnitude 100×). **A** control *Pseudomonas aeruginosa* PCM 2720 biofilm, **B** *Pseudomonas aeruginosa* PCM 2720 after bacteriophage F8 crude suspension treatment, **C** *Pseudomonas aeruginosa* PCM 2720 biofilm after purified bacteriophage preparation treatment

The results of biofilm degradation were confirmed by visualization under a light microscope with crystal violet staining. Figure 4 presents the differences in the coverage of the slides formed by *Pseudomonas aeruginosa* monolayer-untreated control cells (Fig. 4A), cells treated with a crude lysate (Fig. 4B) and cells treated with the purified preparation (Fig. 4C). The observed difference between the control and treated biofilms was significant and agreed with the colony counting assay results (Fig. 3).

### Effect of Serum on the Stability of the Obtained F8 Bacteriophage Preparations

**Fig. 5:**
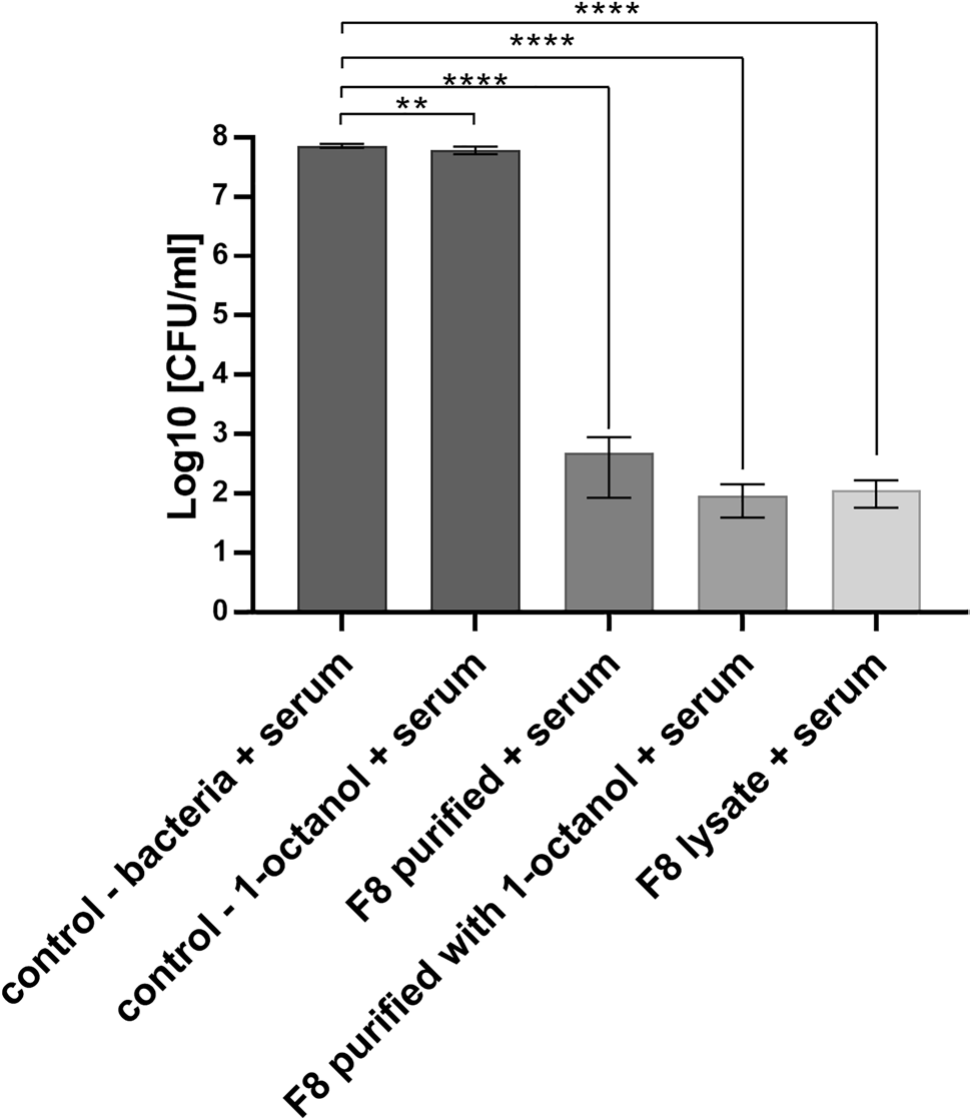
Antibacterial activity of different F8 phage preparations against *Pseudomonas aeruginosa* PCM 2720 in the presence of human serum. **p value < 0.005; ****p value < 0.0005

In the last experiment, the activity of the purified and stabilized phage preparation was evaluated in the presence of human-pooled serum. This study was performed as an introduction to in vivo conditions and to evaluate the possible influence of serum components on phage particles. Moreover, we would like to check whether the bacteriophage and active serum applied together on biofilm can act more effectively than they are applied alone. For this purpose, human-pooled serum was added to bacteria incubated with various F8 bacteriophage preparations, and after 2 h, the suspension was plated on agar plates. Figure 5 summarizes the results of this experiment. All the following controls were used for the experiment: bacteria alone, bacteria with serum, and bacteria with 1-octanol. Additionally controls with heat-inactivated serum were performed and were presented in Supplementary Material to this article (Fig. 2 sup). The results clearly show that the number of bacteria in the presence of human serum did not decrease significantly. Bacteria incubated with bacteriophage F8, both with and without the addition of 1-octanol, significantly reduced the number of bacteria in the medium. The aim of the experiment was to demonstrate the preservation of lytic activity in the obtained bacteriophage preparations against *Pseudomonas* in the presence of pooled human serum and to verify whether prior treatment with bacteriophage has the potential for more effective elimination of bacteria by components of the serum or whether the serum components can inhibit phage activity to kill bacterial cells in this in vitro model. Since the lowest number of bacteria was detected in all preparations treated with active serum and F8 phage, it can be initially concluded that preincubation with a purified and stabilized F8 bacteriophage preparation may support serum activity in the killing and elimination of *P. aeruginosa*. Additionally, the results obtained for the control where heat-inactivated serum and F8 phage support these findings because the efficacy of the bacterial lysis is compared to the phage alone (Fig. 2 sup). All the differences between the samples treated with active serum and F8 phage and controls without phages are statistically significant according to the Fig. 5. Further research in a cell culture or animal model of infection will allow for a better understanding of this issue.

## Discussion

Previous studies have shown that the efficacy of phage therapy depends on the phage concentration delivered at the site of infection. The use of phage preparations without a defined formulation in a clinical trial or treatment is unacceptable. One of the most important problems faced by the implementation of bacteriophage therapy as a standard treatment strategy is the microbiological purity of these preparations and the long-term preservation of their antibacterial activity. The preservation of lysates at 4 °C, freezing (− 80 °C or liquid nitrogen), and preservation of lyophilized phages are commonly used methods for long-term phage storage. Low temperature can significantly decrease the chances of contamination resulting from serial passages. However, a problem associated with preservation at low temperatures is a decrease in bacteriophage titer with time [24]. The preservation of purified phage preparations seems to be a complex issue. In the case of the lysate, some of the components coexisting with the phage could act as protectants. Lysates consist of bacteriological medium compounds and products of the lysis of bacterial cells, such as lipids or proteins, which stabilize phage particles, but their removal is important for bacteriophage applications (some of the bacterial cell components are strong immunogens and should be removed from the final formulation). Only well-purified phage solutions that are free of endotoxins and bacterial cell breakdown products can be used for therapy. Previously, we designed a phage extraction method using 1-octanol [19] and observed that trace residues of 1-octanol in the bacteriophage preparation after extraction allowed longer storage of the purified preparations while maintaining antibacterial activity. This was the reason for our further research, i.e., checking the possibility of using 1-octanol as a preservative of purified phage preparations. As mentioned before, the hydrophobic fatty alcohol 1-octanol is used in the cosmetic industry and is most importantly approved for human use by the FDA. This is undoubtedly an advantage of 1-octanol and could lead to the development of a novel method for the long-term storage of bacteriophage formulations. It was also demonstrated that the stability of enzymes in nonaqueous media may be enhanced compared to that in water. Enzymes suspended in organic solvents display altered activity and specificity [25]. It was proven that proteins dissolved in nonpolar solvents may demonstrate thermal stability. In addition, a stabilizing effect of 1-octanol on insulin has already been observed. In 1-octanol, the native structure of the protein appeared to be maintained, and thermal denaturation was significantly retarded. Additionally, chemical stability was predicted [26].

Our research showed that the addition of 1-octanol has a positive effect on the preservation of high phage titers. We performed a series of experiments evaluating the stability of 3 F8 phage preparations under different environmental conditions: (1) phage lysate, (2) purified phage preparation, and (3) purified phage preparation stored with 1-octanol addition. The best results were observed for the crude lysate of the F8 phage and for the purified phage with 1-octanol addition. Strong variability between phage preparations was observed, with a purified phage F8 being unstable at all 3 tested temperatures. 1-octanol improved storage at 4 °C and 40 °C, but the opposite effect was observed at − 20 °C, where phage activity was lowest after 40 days of storage. Crude lysate cannot be used in therapy and was used to compare bacteriophage activity before and after the purification process. Because *Pseudomonas* bacteria have a strong ability to form biofilms, we investigated the ability of the obtained bacteriophage preparations to eradicate biofilms. The results showed that a purified stabilized preparation allows for better biofilm reduction. Elimination of impurities remaining in the crude lysate likely increases the bioavailability of the phage particles to specific bacteria. The presence of 1-octanol in bacteriophage preparations can be important for biofilm destabilization because it can affect the hydrophobicity of the environment. This increase can be correlated with the increased possibility of bacteriophage penetration into the biofilm structure, which results in more effective destruction of bacterial cells. Alternatively, it can be correlated with reduced adhesion of bacterial cells to the biofilm structure. There are reports about the use of superhydrophobic nanocoatings as a strategy for preventing the formation of bacterial biofilms. Superhydrophobic surfaces can reduce the adhesion of microbial cells through their antifouling properties [27].

Additionally, we evaluated the activity of the obtained phage preparations in active human serum. All tested combinations seemed to be infective. An important observation is the lack of a negative effect of serum on the antibacterial activity of all the tested bacteriophage preparations, which means that all the tested preparations remained stable. Notably, the problem of bacteriophage elimination from the body is known and explored [11]. Bacterial viruses are not inert to the immune system and should be taken into account when selecting doses of bacteriophages used in clinical trials. Research indicates that the immune status of the body is crucial for the success of phage therapy. On the one hand, some reports have shown the influence of human plasma on bacteriophage infectivity reduction. The authors hypothesize that this effect is correlated with plasma proteins, which, by binding to the bacterial surface, mask the receptors recognized by bacteriophages [28]. On the other hand, Tang-Siegel showed that a pseudolysogenic bacteriophage could be switched to a lytic state, resulting in bacterial lysis in response to human serum components [29]. These experiments show that knowledge about the use of bacteriophages in the clinic should be increasingly developed to develop appropriate treatment protocols. Human serum components certainly have an impact on phage particles and could depend especially on the phage but also on the immunological state of the treating patient.

Although phages can be excellent tools in the fight against antibiotic-resistant bacteria, some issues need to be solved. The first and most important consideration is the safety of using phage preparations and their strictly defined composition. In addition, the generation of resistance to phages by bacteria and the reaction of the immune system to phages and their residues should be investigated [30].

## Conclusion

In conclusion, the stabilizing effect of 1-octanol on the storage conditions of the purified F8 bacteriophage preparation was demonstrated. In addition, the efficiency of the purified F8 bacteriophage in the eradication of biofilms formed by *Pseudomonas aeruginosa* was confirmed. All tested combinations of phage preparations remained active in the presence of human serum and infected *P. aeruginosa* cells. Moreover, we observed better killing efficacy when phage was applied with serum on the biofilm compared to the results where serum and phage were applied alone. The obtained results can be an introduction to broader tests on other groups of bacteriophages to confirm the possible universality of the procedure and to the standardization of obtaining bacteriophage preparations, which will allow for their subsequent use in the clinic.

## Supplementary Information

Below is the link to the electronic supplementary material.

Supplementary file1 (DOCX 363 KB)

## Data Availability

Available upon reasonable request.
