# Supplementary material for: The Hydrophobic Stabilization of Pseudomonas aeruginosa Bacteriophage F8 and the Influence of Modified Bacteriophage Preparation on Biofilm Degradation
Source: Curr Microbiol. 2024 Sep 22;81(11):370. doi: 10.1007/s00284-024-03896-2 (PMC11417074; doi:10.1007/s00284-024-03896-2)
Supplement: Supplementary file 1 — Supplementary file1 (DOCX 363 KB) [file 284_2024_3896_MOESM1_ESM.docx]

**Supplementary Information**

**The hydrophobic stabilization of *Pseudomonas aeruginosa* bacteriophage F8 and the influence of the modified bacteriophage preparation on the biofilm degradation**

Bożena Szermer-Olearnik^1#^, Karolina Filik-Matyjaszczyk^1^, Jarosław Ciekot^1^, Anna Czarny^1^

1 Hirszfeld Institute of Immunology and Experimental Therapy, Polish Academy of Sciences, 12 R. Weigl St, Wroclaw, 53114, Poland

#Corresponding author

Bożena Szermer-Olearnik

E-mail: [bozena.szermer-olearnik@hirszfeld.pl](mailto:bozena.szermer-olearnik@hirszfeld.pl)

ORCID: 0000-0002-1451-4227

Fig. 1 sup Dynamic light scattering measurement of the purified F8 bacteriophage repeated 3 times. Z-Average 116,7 nm, PdI value 0,209.


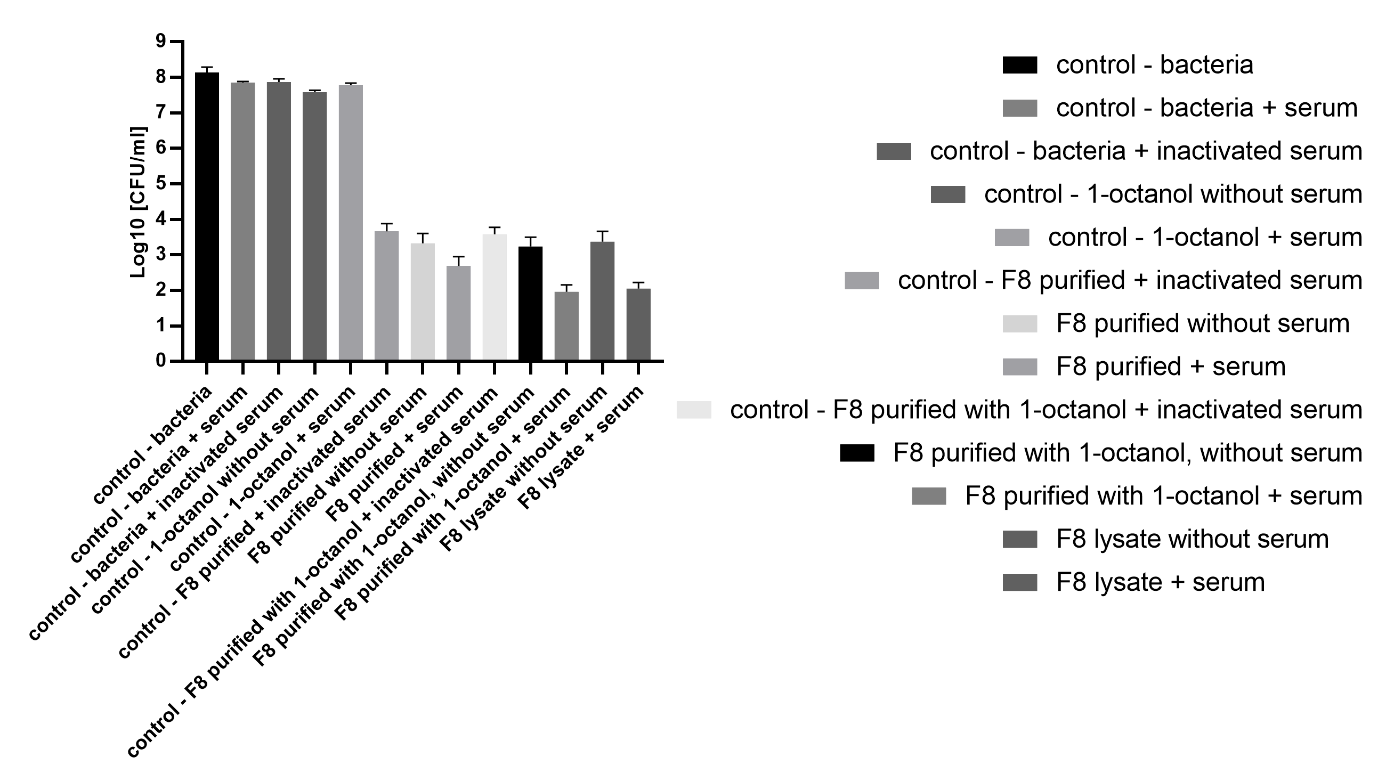


Fig. 2 sup Antibacterial activity of different phage preparations in the presence of human serum.
